# Electron-Impact Resonances of Anthracene in the Presence of Methanol: Does the Solvent Identity Matter?

**DOI:** 10.1021/acs.jpclett.5c01750

**Published:** 2025-07-11

**Authors:** Aude Lietard, Jan R. R. Verlet

**Affiliations:** † Department of Chemistry, 3057 Durham University, Durham DH1 3LE, United Kingdom; ‡ J. Heyrovský Institute of Physical Chemistry, Czech Academy of Sciences, Dolejškova 3, 18223 Prague 8, Czech Republic

## Abstract

Electron impact resonances of neutral molecules can be probed using 2D photoelectron spectroscopy of their radical anions, with a core advantage of being able to introduce solvent molecules in a systematic manner through clustering. This approach has been employed previously to probe the effect of water molecules on the resonances of anthracene. Here, we extend this study to probe the resonances of anthracene in the presence of methanol. We find that the nature of the solvent has little impact on the resonances from the perspective of the anion. Only the electron affinity is observed to increase, which corresponds to a concomitant decrease in resonance energy as viewed from a free electron impacting the anthracene-methanol cluster. For a critical cluster size, *n*_critical_, the lowest resonance becomes a bound state and the mechanism for electron loss switches from a prompt autodetachment process to a statistical thermionic emission process. We posit that the identity of a general solvent molecule only impacts the stabilization of the resonances of anthracene, which in turn affects the overall decay mechanism and *n*_critical_, but the inherent resonance dynamics of anthracene is unaffected by the solvent.

## Graphical abstract

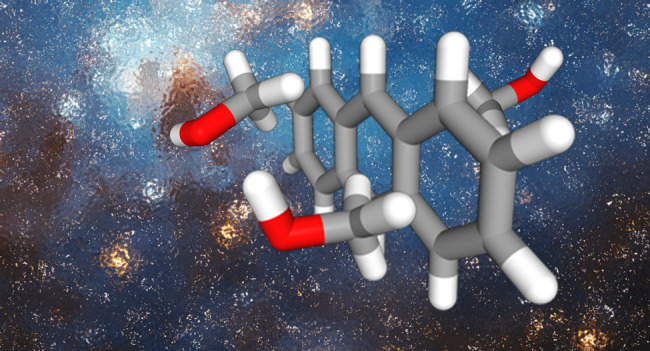

Polycyclic aromatic hydrocarbon (PAH) molecules are common in research areas ranging from medicinal chemistry to atmospheric sciences and from astro-chemistry to molecular electronics.^1–7^ For the latter in particular, it is the redox properties of the PAH that determine its potential for applications in molecular electronics.^4,5^ These redox properties are also linked to the availability of excited states of the radical anions and cations. For example, radical anion excited states of a molecule can enhance its electron accepting ability.^8–11^ The electron accepting ability also has important implications in the interstellar medium, where PAHs in dense molecular clouds are thought to be the main carrier of negative charge rather than electrons.^12–14^ Indeed, it is expected that charged PAH species contribute to the Diffuse Interstellar Bands (DIBs),^15–17^ and several cyano-substituted PAH molecules have been observed through radioastronomy,^18–21^ suggesting that other PAHs including species such as neutral anthracene is likely present in the interstellar medium. The availability of electronic states of the anion that lie in the continuum, so-called electronic resonances,^22,23^ are essential in anion formation, where competition between electron loss (autodetachment) and internal conversion, dictates the yield of metastable anion formation.^8–11^ However, in many instances, a PAH is not isolated but is surrounded by other molecules.^7^ This then leads to the natural question: how do solvent molecules affect the electronic resonances?

The above question has recently been addressed using 2D photoelectron spectroscopy,^24–26^ where photoexcitation from the anion ground state can directly access the electronic resonances. 2D photoelectron spectroscopy has been exploited particularly to probe the electronic resonances of PAHs,^27–30^ in part because both the anion and neutral have similar structures, so that the electron-neutral collision that anion photoexcitation aims to emulate has a stronger correspondence, while appreciating that electron attachment and photodetachment can have differing cross sections.^26^ The key advantages of photoexcitation over electron excitation is that dynamics can be probed, as shown for electronic resonances of PAHs,^10,31^ and that mass-selection is possible prior to investigation, allowing one to incrementally add individual solvent molecules as a molecular cluster.^32–35^ The effect of hydration was first demonstrated for anthracene,^32^ which was shown to have a minimal impact on the location of the excited states in terms of photoexcitation energies (i.e., from the perspective of the anion).^32^ However, when viewed from the perspective of an electron impacting on anthracene, then the electronic resonance energies decrease with increasing hydration (by an amount equal to the increase in electron affinity of the cluster). The same overall trends have been observed for other PAHs such as pyrene,^33^ for N-substituted PAHs,^32^ and for more strongly interacting molecules with water such as the nucleobases uracil^35^ and thymine.^36^ In all cases to date, the solvent has been water. We now consider whether the same conclusion can be drawn with another solvent. Here, we consider the effect of methanol solvent molecules on the electronic resonances of anthracene.

Anthracene is an ideal test case as it has been extensively studied as the smallest linear PAH with a positive adiabatic electron affinity of 0.532(3) eV.^37,38^ Anthracene resonances have been studied computationally,^27,39–42^ and by experimental means including electron scattering experiments^43,44^ and by photoelectron spectroscopy of the corresponding anion^38,45–49^ and its 2D variant.^27–30^ The latter is particularly useful as dynamics taking place on the electronic resonances alter the outgoing autodetached photoelectron spectrum; scanning photoexcitation energies over resonances while measuring their corresponding photoelectron spectra in a 2D manner can therefore offer direct insight of resonance dynamics.^26^ It is analogous to 2D electron energy loss spectroscopy^50–53^ but with the major advantage that the target is charged and can thus be mass-selected.

The 2D photoelectron spectra of cold C_14_H_10_^–^(MeOH)*_n_* with *n* = 0–3 are shown in Figure 1 in the range 1.00 ≤ *hv* ≤ 3.00 eV for *n* = 0 and 1 (Figure 1a,b); 1.05 ≤ *hv* ≤ 3.00 eV for *n* = 2 (Figure 1c) and 1.40 ≤ *hv* ≤ 3.00 eV for *n* = 3 (Figure 1d). The step-size in *hv* for all 2D photoelectron spectra was 0.05 eV. The PAH clusters are formed in a molecular beam expansion that typically have internal temperatures of 10s K, similar to the temperature in dense molecular clouds.

**Figure 1:**
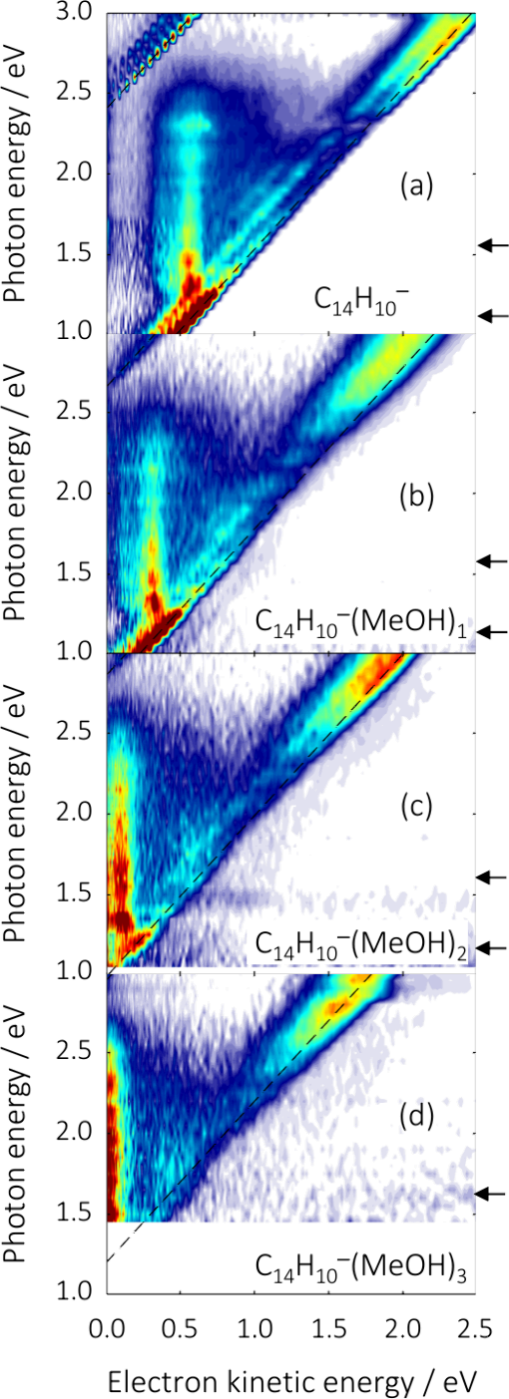
2D photoelectron spectra of C_14_H_10_^–^(MeOH)*_n_* with *n* = 0 (a), *n* = 1 (b), *n* = 2 (c), and *n* = 3 (d). Dashed diagonal lines indicate the direct detachment channels: S_0_ + e^–^ and T_0_ + e^–^. The arrows indicate the location of the lowest energy anion resonances.

The 2D photoelectron spectrum of C_14_H_10_^–^ in Figure 1a has been discussed in detail elsewhere.^27,29^ Briefly, diagonal signals with unit gradient correspond to direct detachment from the anion.^26^ There are two such signals. The one with the highest eKE corresponds to detachment leaving the neutral in the ground electronic state, S_0_ + e^–^, with the observed structure corresponding to the Franck–Condon profile of the detachment.^27,29^ This direct detachment signal intercepts the *hv*-axis at 0.53(1) eV, which corresponds to the electron affinity of C_14_H_10_ (previously determined to be 0.532(3) eV^38^ through higher resolution methods^54^). A second diagonal signal can be seen with *hv* intercept at 2.40(1) eV, which corresponds to direct detachment leaving the neutral in its first electronically excited state: T_1_ + e^–^. Dashed lines are included in the 2D photoelectron spectra to indicate these diagonal features. The difference between the intercepts of both features corresponds to the term energy of the T_1_ state which is 1.87(1) eV, previously determined to be 1.872(3) eV^38^ at higher resolution).

There are also features that are not diagonal in the 2D photoelectron spectrum of C_14_H_10_^–^. These come about from the excitation of resonances; dynamics taking place on the potential energy surface of the resonance lead to a redistribution of the total energy into internal degrees of freedom of the molecule such that the outgoing photoelectron leaves with less kinetic energy.^26^ This is most clearly visible in the excitation range 1.1 < *hv* < 2.5 eV, where a number of resonances are in fact contributing as described previously and the reader is referred to those works for additional details.^27,29^ The peak kinetic energy of this feature remains fairly constant with eKE ∼ 0.6 eV over the *hv* < 2.5 eV range. These features are a very sensitive (albeit indirect) measure of the resonance dynamics. Based on previous work, the location of the two lowest energy resonances^27,29^ are indicated by arrows in Figure 1a. The main purpose of the current work is to address how solvation by methanol affects the observed dynamics of the resonances in C_14_H_10_^–^ and how this differs for clustering to water molecules.

Figure 1b shows the 2D photoelectron spectrum of C_14_H_10_^–^(MeOH)_1_. The same overall features are observed with or without a MeOH molecule present. Specifically, two direct detachment channels are seen as diagonal features. However, these are offset so that their onsets are at higher *hv*. Specifically, the *hv*-intercept of the S_0_ + e^–^ channel has increased to 0.78(1) eV and similarly the T_1_ + e^–^ to 2.67(1) eV, amounting to an increase of ∼ 0.26 eV on average compared to the naked C_14_H_10_^–^. This increase in detachment energy arises from the stronger binding between the C_14_H_10_^–^ and the polar MeOH than the final neutral ground state, where the interaction is between neutral C_14_H_10_ and MeOH. For reference, the increase in energy upon the clustering of a single H_2_O to C_14_H_10_^–^ is 0.22 eV.^32^

In addition to the diagonal features, the indirect autodetachment features seen for the bare C_14_H_10_^–^ are also observable for C_14_H_10_^–^(MeOH)_1_. The photoelectron features with constant eKE visible in the range 1.1 < *hv* < 2.5 eV for C_14_H_10_^–^ are clearly also present for C_14_H_10_^–^(MeOH)_1_ and over the same excitation range. Indeed, the onset of this indirect feature is almost identical between the two (horizontal arrow in Figure 1), while the peak kinetic energy of the autodetachment feature has red-shifted to eKE ∼ 0.3 eV compared to C_14_H_10_^–^.

Figure 1c shows the 2D photoelectron spectrum of C_14_H_10_^–^(MeOH)_2_, which reveals similar changes of features as going from bare C_14_H_10_^–^ to C_14_H_10_^–^(MeOH)_1_. The electron binding energy has increased to 0.99(2) eV for the S_0_ + e^–^ channel and to 2.87(2) eV for the T_0_ + e^–^ channel, corresponding to an increase of 0.20 eV, relative to C_14_H_10_^–^(MeOH)_1_. Evidence of autodetachment and, therefore, resonance dynamics are also clearly observed. The onset energy (*hv*) of the lowest-energy resonance has not shifted appreciably upon the addition of a second MeOH. The indirect autodetachment features are very similar and still span the range 1.1 < *hv* < 2.5 eV, but have red-shifted further to eKE ∼ 0.1 eV (a redshift of about 0.2 eV compared to C_14_H_10_^–^(MeOH)_1_).

Finally, Figure 1d shows the 2D photoelectron spectrum of C_14_H_10_^–^(MeOH)_3_, and continues the same trends. The electron affinity has increased further to 1.2(1) eV for the S_0_ + e^–^ channel. The error has increased substantially as the signal levels near threshold are much poorer compared to the smaller clusters, which is inhibiting the more accurate determination of the onset. The T_0_ + e^–^ channel is now out of the range probed in the current experiment. The energetic onset (*hv*) of the lowest-energy resonance is no longer identifiable, because the electron affinity of the C_14_H_10_(MeOH)_3_ has exceeded this onset, suggesting that the resonance has become a bound excited state. Nevertheless, there is clear evidence that similar dynamical processes are still taking place. The indirect autodetachment feature now appears to peak near zero kinetic energy but still spans up to the same limit of *hv* < 2.5 eV. Figure 2 shows a typical photoelectron spectrum at *hv* = 2.30 eV which highlights this low energy autodetachment peak, the shape of which is an exponentially decaying function with eKE, which is consistent with a mechanism in which the electron is detached from the cluster statistically (typically over many microseconds).^55–57^

**Figure 2:**
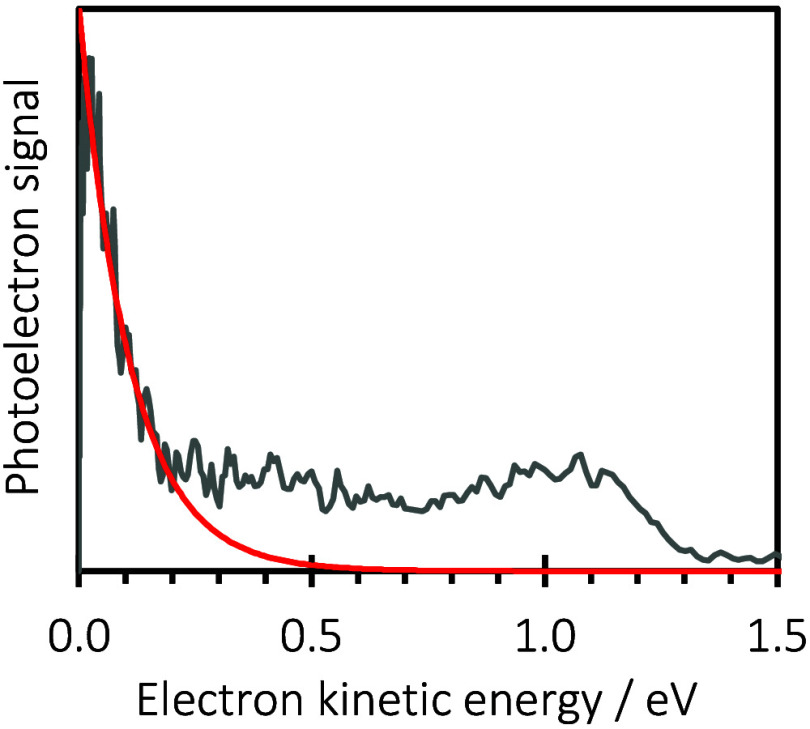
1D photoelectron spectra of C_14_H_10_^–^(MeOH)_3_ at *hv* = 2.30 eV. Red line is a exponentially decaying function highlighting the statistical origin of the low eKE feature (thermionic emission).

In summary, we observe that, upon incremental solvation, the electron affinity of C_14_H_10_ increases and the S_0_–T_1_ energy gap remains constant. The energy of the autodetachment peak decreases in eKE by a similar amount as the electron affinity increases, which is consistent with a view that the resonance energies are decreasing relative to the S_0_ neutral ground state.^32^

The overall trends observed indicate that the electronic structure and resonance dynamics of C_14_H_10_^–^ are not impacted significantly by the presence of the solvating MeOH molecules. In Table 1, we compare the onsets of direct detachment channels observed for C_14_H_10_^–^(MeOH)*_n_* with those for C_14_H_10_^–^(H_2_O)*_n_* for similar *n*,^32^ as well as the peak position of the autodetachment peak. Comparing binding energies between MeOH and H_2_O shows that clustering leads to almost identical changes, despite the differing solvent molecule. One might anticipate a similar stabilization of the anion by clustering a hydrophobic anion to either MeOH or H_2_O given the similar dipole moments of the solvent molecule (μ = 1.70 and 1.85 D for MeOH and H_2_O, respectively). The resonance dynamics that are captured by the peak of the autodetachment emission, suggest that the dynamics associated with the autodetachment from the resonances is also not impacted in a noticeable manner. This is perhaps more surprising given that the solvent interaction to excited states of an anion might be expected to be more sensitive as the electron is more weakly bound, and certainly, the potential energy landscapes might be expected to be impacted differently. This expectation is not observed here.

**Table 1:** Onset of the Direct Detachment Channels Leading to S_0_ + e^–^ and T_0_ + e^–^ for Both C_14_H_10_^–^(MeOH)*_n_* and C_14_H_10_^–^(H_2_O)*_n_* ^a^

	C_14_H_10_^–^(MeOH)*_n_*			C_14_H_10_^–^(H_2_O)*_n_*		
*n*	S_0_ + e^–^	T_0_ + e^–^	AD	S_0_ + e^–^	T_0_ + e^–^	AD
0	0.53(1)	2.40(1)	0.6	0.53(1)	2.40(1)	0.6
1	0.78(1)	2.67(1)	0.3	0.75(1)	2.64(1)	0.3
2	0.99(2)	2.87(2)	0.1	0.99(2)	2.89(2)	0.1
3	1.2(1)	n/a	n/a	1.16	3.03	n/a

a Also shown is the electron kinetic energy peak for the lowest energy autodetachment (AD) feature for both solvent species. All energies are in eV.

Our observations that resonance excitation energies are not impacted by the extent of solvation is similar to observations of C_14_H_10_^–^(H_2_O)*_n_* and other radical PAH anions including pyrene,^33^ acridine,^32^ phenazine,^32^ and the nucleobases uracil^35^ and thymine.^36^ We now extend this observation to note that the nature of the solvent is also not impacting the excitation energy to the resonance (although we caveat this by noting that this is only for two data points – water and methanol).

Given the similarity between the resonance dynamics and positions for MeOH and H_2_O, we can extend the conclusions from previous work on C_14_H_10_^–^(H_2_O)*_n_* to C_14_H_10_^–^(MeOH)*_n_*. Electrons emitted at very low eKE, peaking at eKE = 0, are indicative of emission via a statistical process (thermionic emission^55–57^ ). Such processes come about when the ground electronic state is recovered following excitation to a resonance. For example, if a resonance can decay to the ground state through some internal conversion process before the electron is lost by autodetachment, then the ground state anion will have sufficient energy to statistically boil-off an electron (or solvent molecule), which typically takes place over many microseconds and produces a characteristic Boltzmann eKE distribution peaking at eKE = 0 eV.^26^ Such emission is seen in Figure 1d for C_14_H_10_^–^(MeOH)_3_ and not for the smaller clusters, where the autodetachment feature peaks at higher eKE. These observations are identical to those for C_14_H_10_^–^(H_2_O)*_n_*, where we conclude that for *n* = 3, the lowest resonance becomes a bound electronic state.^32^ Excitation to resonances in C_14_H_10_^–^(H_2_O)*_n_* are able to rapidly decay by internal conversion to nearby electronic states. For *n* < 3, those electronic states are resonances and the electron is emitted by autodetachment. For *n* ≥ 3, the lowest excited state is bound and can no longer decay by autodetachment. Instead, it returns to the ground state and undergoes thermionic emission. The same appears to be the case for C_14_H_10_^–^(MeOH)*_n_*.

We now consider how conclusions from C_14_H_10_^–^(H_2_O)*_n_* and their relevance to electron processes in dense molecular clouds can be extended based on our current observations. The energy of electrons in such environments is thought to be thermalized with the background (∼10 K),^58^ suggesting eKE of incoming electrons near 0 eV, so that resonances will be available for *n* ≥ 2 clusters. Models that predict that PAHs are a major sink for electrons in dense molecules clouds consider PAH molecules with <30 C atoms to be too small to be able to attach electrons efficiently.^59–62^ Hence, C_14_H_10_ would not be expected to attach electrons effectively. For the bare C_14_H_10_, this may be the case, but it is also expected that molecules can condense onto PAHs (and PAHs can condense onto grains), given the cold (∼10 K) environment in a nebula.^6,7^ The condensation of a just 3 water molecules was argued sufficient to enhance the PAHs electron capture ability^32^ as discussed above. However, we now extend this by noting that the solvent molecule need not be H_2_O; it can just as well be MeOH (as demonstrated here). Indeed, it is likely that a combination of the two (i.e., mixed C_14_H_10_^–^(MeOH)*_n_* (H_2_O)*_m_*) would behave similarly (i.e., *n* + *m* ≥ 3 would lead to a bound state). Note also that, at these incident energies, the solvent molecules considered here have no resonances and therefore, from an electron impact perspective, the solvent molecules do not present a barrier to electron attachment (certainly not for the relatively small clusters considered here).

Extending the above arguments further, we posit that the chemical nature of the solvent is unimportant, and only its physical properties matter. The role of the solvent is to increase the electron affinity of the PAH, which stabilize the resonances relative to the neutral ground state by a similar amount. Water and methanol increase the electron affinity by a significant amount (>0.2 eV per molecule for the first few solvent molecules); others might increase the electron affinity more or less, depending on the interaction. Solvent molecules such as CO, H_2_, and CO_2_ are not likely to increase the electron affinity by a large amount on account of their low (or nonexistent) dipole moment compared to MeOH or H_2_O; more solvent molecules (i.e., *n* > 3) are likely required in such cases. On the other hand, solvent molecules such as ammonia and ethanol are likely to be similar to MeOH and H_2_O. Regardless of the molecules, we anticipate that the resonance dynamics of the C_14_H_10_^–^ core remain unchanged. At present, this is a just a hypothesis, which future work probing different solvent clusters will aim to explore.

The above arguments do consider the solvent interaction between a nonpolar PAH, while in the interstellar medium, PAHs commonly contain heteroatoms and functional groups. For example, several cyano-containing PAHs have been observed. Our previous study on the effect of H_2_O solvation on resonances of anthracene additionally considered the singly- and doubly substituted central C atoms with N atoms. While, both acridine and phenazine have differing electronic structures and electron affinities, both showed very similar changes to the addition of H_2_O, suggesting that the inclusion of the heteroatom (which for acridine also induces a permanent dipole) in the PAH does not lead to a significant change upon hydration. While we have yet to do similar experiments with MeOH as a solvent, we expect a broadly similar outcome in that case.

In conclusion, 2D photoelectron spectroscopy of C_14_H_10_^–^(MeOH)*_n_* with *n* = 0 – 3 has been used to probe the electronic resonances and their dynamics of C_14_H_10_^–^ in the presence of methanol molecules. We find that, upon clustering, the excitation energy from the ground electronic state of the radical anion to the resonances do not change significantly and neither do the overall resonance dynamics, nor the S_0_–T_1_ energy gap of the neutral. However, the electron affinity of the cluster (i.e., the D_0_–S_0_ energy gap) is increasing due to the stronger interaction between the solvent and the anion compared to the neutral. The overall consequence is that the resonances are being stabilized with respect to the neutral ground state. For *n* = 3, the lowest energy resonance becomes a bound state and thermionic emission is observed. These observations are compared to similar experiments performed on C_14_H_10_^–^(H_2_O)*_n_* and found to be almost identical in all regards. We conclude that the identity of the solvent molecule, be it water or methanol, is not important in the intrinsic resonance dynamics of C_14_H_10_^–^ in the solvent-clustered C_14_H_10_^–^. However, we recognize that methanol and water are similar, especially in terms of their dipole moment, which is the dominant factor in stabilizing the anion (i.e., charge-dipole interaction). We anticipate that differing solvent molecules with differing dipole moments will stabilize the anion differently and therefore, the critical size, *n*_critical_, at which the lowest energy resonance becomes a bound state may vary for different solvent molecules. For water, methanol, or a combination thereof, *n*_critical_ = 3. We posit that, generally, the chemical identity of the solvent molecule has little effect on the resonance dynamics, and only the physical properties of the solvent (dipole moment) determines *n*_critical_. We discuss our findings in the context of electron capture by PAHs in dense molecular clouds.

## Methods

The experiment has been described in detail elsewhere^63^ and only a brief outline is provided. Solid anthracene (Sigma-Aldrich) was heated to 180 °C in an Even-Lavie valve.^64^ The anthracene vapor was expanded through the pulsed valve into vacuum using Ar as a carrier gas (5 bar) with a drop of methanol placed in the backing line. The resultant molecular beam containing anthracene, methanol and Ar was then intersected by an electron beam (300 eV) near the throat of the expansion. This formed anthracene anions, C_14_H_10_^–^ along with its clusters, (C_14_H_10_)*_n_* ^–^, and anthracene-methanol clusters, C_14_H_10_^–^(MeOH)*_n_*. These clusters are generally cold although defining an absolute temperature is difficult because the energy is not evenly distributed (rotational temperature is typically lower than vibrational). Nevertheless, vibrational temperatures are typically on the few 10s K, which is consistent with the typical temperatures in a dense molecular cloud. Mass-selection was achieved using a Wiley–McLaren time-of-flight spectrometer^65^ and the ion packet of choice was intersected by nanosecond laser pulses from a tunable Nd:YAG pumped optical parametric oscillator. The resulting photodetached electrons were analyzed using a velocity map imaging spectrometer^66,67^ and images were reconstructed using the polar onion-peeling algorithm^68^ to offer photoelectron spectra (and angular distributions that are not discussed here). The electron kinetic energy (eKE) scale was calibrated using the known photoelectron spectrum of I^–^. Photoelectron spectra have a resolution of ΔeKE/eKE ∼ 3%.

## Supplementary Material



## Data Availability

Raw photoelectron spectra used to generate the 2D photoelectron spectra are available at https://doi.org/10.5281/zenodo.15746443.

## Notes

Transparent Peer Review report available (PDF)
