# Supplementary material for: Electron-Impact Resonances of Anthracene in the Presence of Methanol: Does the Solvent Identity Matter?
Source: J Phys Chem Lett. 2025 Jul 11;16(29):7307–12. doi: 10.1021/acs.jpclett.5c01750 (PMC12302206; doi:10.1021/acs.jpclett.5c01750)
Supplement: Supplementary file 1 [file jz5c01750_si_001.pdf]

jz-2025-01750v.R1

Name: Peer Review Information for "Electron-Impact Resonances of Anthracene in the Presence of Methanol: Does the Solvent Identity Matter?"

First Round of Reviewer Comments

Reviewer: 1

Comments to the Author

Please, see in attached file.

This paper by Aude Lietard and Jan R. R. Verlet entitled “Electron-impact resonances of anthracene in the presence of methanol: Does the solvent identity matter?” reports new important experimental findings on the electronic resonances and their dynamics for anthracene molecular negative ions surrounded by one to three methanol molecules. The results obtained using 2D photoelectron spectroscopy of radical anions are compared to earlier experiments by this group for anthracene surrounded by water molecules. The overall conclusion is that the findings are almost identical despite the nature of the solvent.

(1) The major reported advance consists of the following findings (a) the nature of the solvent has little impact on the electronic structure; the resonance dynamics (in the present case of anthracene surrounded by H2O or MeOH molecules) is unaffected by the solvent, (b) a number of surrounding molecules (a critical cluster size) to stabilize the lowest anion state into a bound region is found to be three as can be observed by the switch from a rapid electron detachment process to a statistical thermionic emission of excess electron which usually appears on microseconds timescale.

(2) The immediate significance consists of possibility to apply the present findings to understand the processes of resonance electron attachment to polycyclic aromatic hydrocarbons (PAHs) in dense molecular clouds. In fact, anthracene can be considered as a model electron acceptor PAH molecule with well-known adiabatic electron affinity; conclusions of the present paper can be extrapolated to other PAH anions including pyrene, acridine, phenazine, as well as the nucleobases (uracil and thymine).

(3) The paper is very accurately written; no typos can be reported as far as I can judge. The only my remark concerns a caption of Fig.1. “Dashed horizontal lines…”. Dashed lines are rather diagonal, not horizontal.

Finally, I recommend publication of this paper in The Journal of Physical Chemistry Letters as is.

Reviewer: 2

Comments to the Author

This is a brief manuscript on cold anthracene anions clustered with 1 to 3 methanol molecules. It builds on a similar study conducted on water cluster anions. In the astrochemistry context, the clusters are important as they act as intermediate cases between free molecules and ices (e.g. H2O, CO2, …). Menthol could be considered as a model organic ice. The interpretation focuses on trends across spectra and comparison with the earlier water anion complexes. It is important to investigate these systems in a systematic manner. The manuscript is well written and logical, and I see the logic in one of their main conclusions ‘Extending the above arguments further, we posit that the chemical nature of the solvent is unimportant, and only its physical properties matter.’. On the other hand, this conclusion is perhaps not very surprising considering that CH3OH and H2O are hydrogen-bonding and involve other inductive & dispersive forces. Anthracene is non-polar. I expect these conclusions to change if the core PAH contains heteroatom sites – perhaps discuss cyano versions that have been observed in space? For example, specific solvent-solute interactions will alter some resonances to a greater degree than others. Overall, the article is probably publishable, subject to some revisions.

It would be useful to highlight in the introduction that small cyano-substituted PAHs are being widely observed in some regions of space through radioastronomy, which supports that neutral anthracene will likely be present (can’t detect with radioastronomy).

I need to see some details on expected temperature/internal energy of the cluster anions. This is important as it helps to inform on phase space the n=2,3 clusters might occupy.

The photoexcitation process to access resonances has been likened to electron capture of a free electron by the same resonances in the neutral + electron system. What can be said about relative cross-sections in electron capture compared with photoexcitation? As more solvents are added, should the electron capture cross-sections as orbitals are blocked? On the other hand, the photoexcitation case does not suffer in the same way. How similar are the solvent electron scattering cross-sections for the relevant electron energies? How broad (in terms of energy) are these resonances, does this change with solvent clustering?

It is hard for me to assess the thermionic emission statement without some example 1D spectra taken from the 2D spectra. On P8: 'These observations are identical to those for $C_{14}H_{10}^{-}(H_2O)_n$, where we conclude that for n = 3, the lowest resonance becomes a bound electronic state28'. Could this apparent thermionic emission signal not be from the ground state, but from internal conversion to an electronic state near the detachment threshold followed by autodetachment?

The article discusses electron attachment in dense molecular clouds. What are expected electron energy distributions in such clouds, and how do these correspond to the photoexcitation energies in the 2D spectra?

Author's Response to Peer Review Comments:

Dear Editor,

Thank you for passing on the reviewer comments. We are delighted with the overall positive view of our work! We would also like to thank reviewer 2 for the useful suggestions that we feel will make the manuscript better. Below, we address the comments and suggestions from the reviewers (in red). We have also included the editorial revisions (see bottom of this document). We have made changes in a word document using the "track-changes" and have also uploaded a clean version of the revised manuscript.

Thank you again and best wishes

Jan

Reviewer: 1

The only my remark concerns a caption of Fig.1. “Dashed horizontal lines…”. Dashed lines are rather diagonal, not horizontal.

We have made this change.

Reviewer: 2

Recommendation: This paper may be publishable, but major revision is needed; I would like to be invited to review any future revision.

Comments:
This is a brief manuscript on cold anthracene anions clustered with 1 to 3 methanol molecules. It builds on a similar study conducted on water cluster anions. In the astrochemistry context, the clusters are important as they act as intermediate cases between free molecules and ices (e.g. H_2O, CO_2, …). Menthol could be considered as a model organic ice. The interpretation focuses on trends across spectra and comparison with the earlier water anion complexes. It is important to investigate these systems in a systematic manner. The manuscript is well written and logical, and I see the logic in one of their main conclusions ‘Extending the above arguments further, we posit that the chemical nature of the solvent is unimportant, and only its physical properties matter.’. On the other hand, this conclusion is perhaps not very surprising considering that CH_3OH and H_2O are hydrogenbonding and involve other inductive & dispersive forces. Anthracene is non-polar. I expect these conclusions to change if the core PAH contains heteroatom sites – perhaps discuss cyano versions that have been observed in space? For example, specific solvent-solute interactions will alter some resonances to a greater degree than others. Overall, the article is probably publishable, subject to some revisions.

The reviewer makes an excellent point. We have actually studied acridine and phenazine previously with water as a solvent (and more strongly interacting molecules such as uracil and thymine), which showed that this did not alter the impact of the

solvation. While we have not done the experiments on these molecules with methanol as a solvent, we can point to this previous work in suggesting a similar observation. We have included this in the discussion.

It would be useful to highlight in the introduction that small cyano-substituted PAHs are being widely observed in some regions of space through radioastronomy, which supports that neutral anthracene will likely be present (can't detect with radioastronomy).

Agreed. We have included a brief discussion on these now.

I need to see some details on expected temperature/internal energy of the cluster anions. This is important as it helps to inform on phase space the n=2,3 clusters might occupy.

The clusters are formed using a molecular beam expansion, which generally produces internally cold species. However, defining an absolute temperature is difficult because the energy is not evenly distributed (rotational temperature is typically lower than vibrational). Nevertheless, vibrational temperatures are typically on the few 10s K, which is consistent with the typical temperatures in a dense molecular cloud. Note that there is no evidence of hot-bands in the photoelectron spectra. We briefly state this in the text and included a discussion on this in the Methods section.

The photoexcitation process to access resonances has been likened to electron capture of a free electron by the same resonances in the neutral + electron system. What can be said about relative cross-sections in electron capture compared with photoexcitation? As more solvents are added, should the electron capture cross-sections as orbitals are blocked? On the other hand, the photoexcitation case does not suffer in the same way. How similar are the solvent electron scattering cross-sections for the relevant electron energies? How broad (in terms of energy) are these resonances, does this change with solvent clustering?

A few questions to unpack: *What can be said about relative cross-sections in electron capture compared with photoexcitation?* The selection rules for electron attachment and photodetachment are different, however, at low energy (as considered here), the cross sections are dominated by dipole operators. So, while cross sections will differ, they often are not as wildly different as one might anticipate. *As more solvents are added, should the electron capture cross-sections as orbitals are blocked?* No, this should not be an issue, and certainly not at the small cluster sizes considered here. Note that electron attachment is possible in bulk solutions (as is common in for example radiation chemistry). *How similar are the solvent electron scattering cross-sections for the relevant electron energies?* There are no solvent resonances in the range considered here – for both H_2O and MeOH, the first resonance is at ~6.5 eV. *How broad (in terms of energy) are these resonances, does this change with solvent clustering?* That is a great

question. We don't know the resonance widths (yet), but do note that the shape of the autodetachment spectra are a signature of the resonance dynamics. The fact that we do not see changes to the autodetachment spectra with solvation, strongly suggests that there is minimal effect on the lifetime of the resonances (width) with solvent clustering. We appreciate that this is a rather indirect measure, but note that a direct measurement of the spectral width of resonances will more than likely be obscured by the presence of the solvent molecules.

We have included some of these considerations in the text, but not all as we felt that some of the discussion would then take away from the core message of the paper.

It is hard for me to assess the thermionic emission statement without some example 1D spectra taken from the 2D spectra. On P8: 'These observations are identical to those for C14H10–(H2O)n, where we conclude that for n = 3, the lowest resonance becomes a bound electronic state28'. Could this apparent thermionic emission signal not be from the ground state, but from internal conversion to an electronic state near the detachment threshold followed by autodetachment?

The reviewer is right that we should have included exemplar 1D photoelectron spectra. We have now done this, which includes an exponentially decaying function to demonstrate that the signal is truly statistical. It is not likely that autodetachment from a resonance would lead to such a spectra shape, for a large part because electronic autodetachment would

The article discusses electron attachment in dense molecular clouds. What are expected electron energy distributions in such clouds, and how do these correspond to the photoexcitation energies in the 2D spectra?

The energy of electrons in dense molecular clouds is thought to be thermalized with the background (10s K), suggesting eKE of incoming electrons near 0 eV. This is of relevance as the for all clusters excluding the bare anthracene, we anticipate resonances to be available at this energy. We have included this consideration in the discussion.

Editorial revisions

1. Please indicate the corresponding author(s) with an asterisk in the author list on the manuscript title page.

Done

2. Please include author names, article titles, journal name, publication year, and at least the first page number for the following incomplete journal references: 2, 53.
Done for reference 2 has a article number. Reference 53 which has now become 56 was correct where R1 is the article number.

Theses: Please include author name, title, institution name, and year for the following incomplete thesis reference: 14.

This is not a thesis but a proceedings paper; we have added the conference proceedings.

3. Include a TOC graphic illustrating the significance of the paper. For TOC guidelines and size requirements, please see the following link: https://eur01.safelinks.protection.outlook.com/?url=https%3A%2F%2Fpubsapp.acs.org%2Fparagonplus%2Fsubmission%2Ftoc_abstract_graphics_guidelines.pdf&data=05%7C02%7Cj.r.r.verlet%40durham.ac.uk%7C99f6e8252b94464388fa08ddb2d6d27c%7C7250d88b4b684529be44d59a2d8a6f94%7C0%7C0%7C638863357911433448%7CUnknown%7CTWFpbGZsb3d8eyJFbXB0eU1hcGkiOnRydWUsIlYiOiIwLjAuMDAwMCIsIlAiOiJXaW4zMiIsIkFOIjoiTWFpbCIsIldUIjoyfQ%3D%3D%7C0%7C%7C%7C&sdata=3UfkT7elWOQyB2oJq6DGyC %2BZfM7MIe1VJUd%2BG0zeZRM%3D&reserved=0? Please label the TOC graphic as "TOC Graphic". A caption describing the TOC graphic is not needed and will not be used.

For more details, please see the Author Guidelines, which can be found here: https://eur01.safelinks.protection.outlook.com/?url=https%3A%2F%2Fpubs.acs.org%2Fpage%2Fjpclcd%2Fsubmission%2Fauthors.html&data=05%7C02%7Cj.r.r.verlet%40durham.ac.uk%7C99f6e8252b94464388fa08ddb2d6d27c%7C7250d88b4b684529be44d59a2d8a6f94%7C0%7C0%7C638863357911455227%7CUnknown%7CTWFpbGZsb3d8eyJFbXB0eU1hcGkiOnRydWUsIlYiOiIwLjAuMDAwMCIsIlAiOiJXaW4zMiIsIkFOIjoiTWFpbCIsIldUIjoyfQ%3D%3D%7C0%7C%7C%7C&sdata=ijCUg6zTYUvDwzntAGcK1Zzc8J%2B%2FN4opEcX4 Iwp0Q5U%3D&reserved=0

Done.
